# Optical Fiber Sensor Based on Localized Surface Plasmon Resonance Using Silver Nanoparticles Photodeposited on the Optical Fiber End

**DOI:** 10.3390/s141018701

**Published:** 2014-10-09

**Authors:** J. Gabriel Ortega-Mendoza, Alfonso Padilla-Vivanco, Carina Toxqui-Quitl, Placido Zaca-Morán, David Villegas-Hernández, Fernando Chávez

**Affiliations:** 1 División de Ingenierías, Universidad Politécnica de Tulancingo, UPT, C.P. 43629 Hidalgo, Mexico; E-Mails: alfonso.padilla@upt.edu.mx (A.P.-V.); carina.toxqui@upt.edu.mx (C.T.-Q.); david.villegas@upt.edu.mx (D.V.-H.); 2 ICUAP, Benemérita Universidad Autónoma de Puebla, BUAP, C.P. 72050 Puebla, Mexico; E-Mails: placido.zaca@correo.buap.mx (P.Z.-M.); fchr172@hotmail.com (F.C.)

**Keywords:** localized surface plasmon resonance, photodeposition, fiber optic sensor, refractive index, silver nanoparticles

## Abstract

This paper reports the implementation of an optical fiber sensor to measure the refractive index in aqueous media based on localized surface plasmon resonance (LSPR). We have used a novel technique known as photodeposition to immobilize silver nanoparticles on the optical fiber end. This technique has a simple instrumentation, involves laser light via an optical fiber and silver nanoparticles suspended in an aqueous medium. The optical sensor was assembled using a tungsten lamp as white light, a spectrometer, and an optical fiber with silver nanoparticles. The response of this sensor is such that the LSPR peak wavelength is linearly shifted to longer wavelengths as the refractive index is increased, showing a sensitivity of 67.6 nm/RIU. Experimental results are presented.

## 1. Introduction

Sensors based on surface plasmon have been widely used to detect biological and chemical analytes, environmental control, and biomedical applications, among others [1–6]. Their main features are fast response, high sensitivity, and the detection is free-label [4,5]. The surface plasmons are coherent oscillations of free electrons at the metal-dielectric interface which are often categorized into two classes: (1) propagating surface plasmons and (2) localized surface plasmons. Propagating surface plasmons are evanescent electromagnetic waves, which are propagating along of a flat metal-dielectric interface and they arise from oscillation of the conduction electrons whereas localized surface plasmons are non-propagating excitations of the conduction electrons of metal nanoparticles. When the oscillation frequency of the conduction electrons within the metal nanoparticles coincides with the light frequency, the resonance condition is obtained giving rise to large extinction coefficients. The localized surface plasmon resonance (LSPR) peak wavelength and its width depends strongly on the composition, size, shape, dielectric environment, and separation distance of the nanoparticles [6,7]. Traditional surface plasmon resonance (SPR) sensors are manufactured in the base of the Kretschmann configuration where a thin noble metal film is coated on a prism, although there are others configurations such as Otto prism coupler, optical waveguides coupler, diffraction gratings, and optical fiber coupler [1]. On the other hand, the LSPR sensors are normally fabricated with noble metal nanoparticles which are deposited on a substrate such as optical fiber. LSPR sensors have additional features such as compact size, electromagnetic immunity, and portability [8]. To fabricate an optical fiber sensor based on LSPR, there are some methods to immobilize nanoparticles on the optical fiber, such as electron-beam lithography [9–11], self-assembly of polyelectrolyte [12,13], and self-assembly [14–16]. Table 1 shows the main features of the methods mentioned above.

In this article, we report the construction of an optical fiber sensor based on LSPR phenomenon using silver nanoparticles. These nanoparticles were immobilized on the optical fiber end by the photodeposition technique. A tungsten lamp along with the optical fiber with nanoparticles and a spectrometer were used to assembly the optical sensor and to detect refractive index in different aqueous media. Our results show that, the LSPR peak wavelength is linearly shifted to longer wavelengths as far the refractive index is increased with a sensitivity of 67.5 nm/RIU.

## 2. Experimental Section

### 2.1. Background: LSPR and Surrounding Medium

The dependence of LSPR peak wavelength with the dielectric function of the surrounding environment can be proven by means the Drude model [6,17,18]

(1) $$\varepsilon_{r}=1-\frac{\omega_{p}^{2}}{\omega^{2}+\gamma^{2}}$$

where *ε*_r_ denotes the real part of the complex dielectric function (*ε*) of the plasmonic material, *ω* is the angular frequency of the radiation, *ω_p_* is the plasma frequency and γ is the damping parameter of the bulk metal. In the visible and near-infrared regions γ ≪ *ω_p_*, so that, the above expression becomes

(2) $$\varepsilon_{r}=1-\frac{\omega_{p}^{2}}{\omega^{2}}$$

The polarizability *α* of a small spherical nanoparticle with a much smaller size than the wavelength of the light is given by

(3) $$\alpha =3\varepsilon_{0}V\frac{\varepsilon -\varepsilon_{m}}{\varepsilon +2\varepsilon_{m}}$$

where *V* is the nanoparticle volume, *ε*_0_ is the free space permittivity, and *ε_m_* is the dielectric constant of the surrounding medium. The plasmon resonance occurs when polarizability attains a maximum, that is, when Equation (3) diverges. Accordingly, the resonance condition is *ε_r_* = −2*ε_m_*.

Using the Equation (2) and the resonance condition, we get the frequency of the LSPR peak which is denoted by *ω_max_* and it is given as follows

(4) $$\omega_{max}=\frac{\omega_{p}}{{\left(2\varepsilon_{m}+1\right)}^{½}}$$

Considering that λ = 2 π*c/ω* and *ε_m_* = *n*^2^, Equation (4) becomes

(5) $$\lambda_{max}=\lambda_{p}\sqrt{2n_{m}^{2}+1}$$

where λ*_max_* is the LSPR peak wavelength and λ_p_ is the wavelength corresponding to the plasma frequency of the bulk metal. Therefore, it is important to note that there exists a linear relationship between LSPR peak wavelength and the refractive index of the surrounding medium.

The sensitivity *S* of a LSPR sensor expressed in nanometers per refractive index unit (nm/RIU) is defined as the change in the LSPR peak wavelength maximum per unit change in the refractive index of the medium and it can be calculated by

(6) $$S=\frac{\Delta \lambda }{\Delta n}$$

### 2.2. Photodeposition of Silver Nanoparticles on the Optical Fiber End

**Figure 1.**
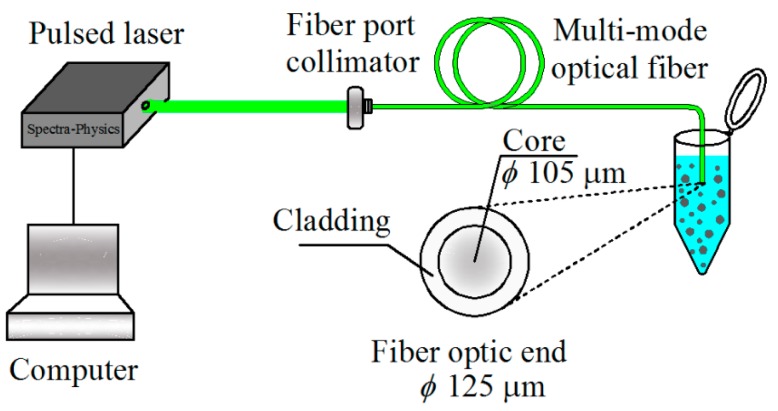
Experimental setup for photodepositing silver nanoparticles on the optical fiber end.

We have implemented the photodeposition technique to deposit silver nanoparticles on optical fiber end using laser light and silver nanoparticles suspend in aqueous environments. In previous works [19], we have realized a theoretical and experimental study about this technique by means of zinc nanoparticles and laser light which travels into a single-mode optical fiber. The origin of the technique is based on the radiation pressure of laser light (absorption and scattering forces) and the force is achieved by particles moving in convection currents (Stokes force) which are provoked by the strong absorption photons in the metal nanoparticles. The mechanism of particle deposition is a well-known phenomenon. Particles close to the core of the optical fiber end are adhered to it. This phenomen is produced by particle interactions through a double layer repulsion and London attraction force [19,20]. With this method is possible to choose the maximum size of nanoparticles adhered on the optical fiber end by means of the laser power. Furthermore, the amount of nanoparticles depends on laser power and the time of the optical fiber submerged into the solution. A pulsed laser emitting at λ = 532 nm with a pulse width <15 ns (Mod. Explorer 532 NM from Spectra-Physics) and a fiber port collimator (PAF-X-2-A from Thorlabs) for coupling a free space laser into a multi-mode optical fiber (FG105LCA from Thorlabs) were used to perform the photodeposition process (see Figure 1). The maximum peak intensity of laser pulse via optical fiber is 385 kW/cm^2^. A power meter (2935T-C from Newport) was used to measure optical power. The multi-mode optical fiber was prepared by removing the coating, after that, it was cleaved and subsequently placed into the solution.

Previously, the seed solution was prepared by mixing 1 c.c. of ethanol and 1 mg of silver nanoparticles with spherical geometry (No. 576832 from Aldrich) whose sizes are smaller than 100 nm.

### 2.3. Measurement Setup

**Figure 2.**
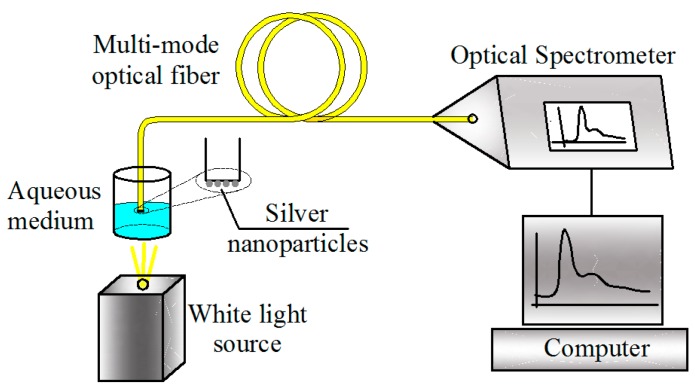
Experimental setup for measuring the refractive index of an aqueous medium.

The optical sensor was composed by a tungsten lamp as a white light source and a spectrometer (Mod. OSM2-400DUV-U from Newport) to measure the absorption/transmission spectra. The optical fiber end was put into the aqueous medium under study, and the absorption spectrum was recorded according to Figure 2. To get the reference spectrum, the optical fiber end without nanoparticles was put into the recipient without liquid and the transmission spectrum was recorded.

## 3. Results and Discussion

**Figure 3.**
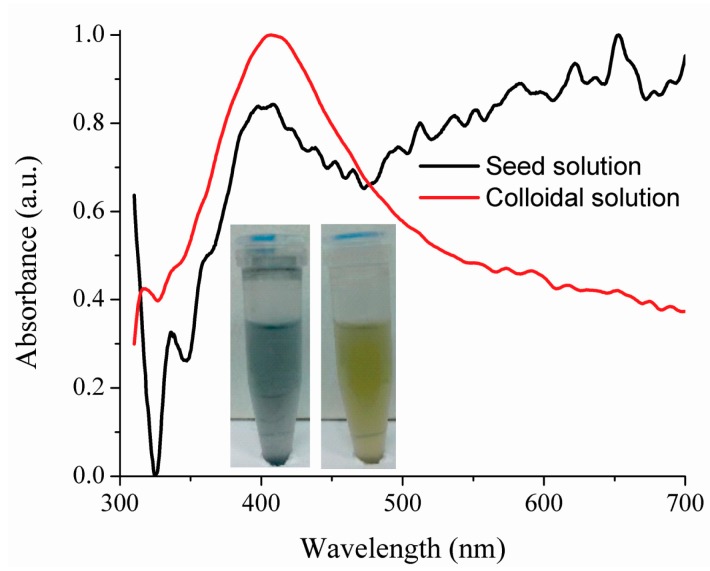
Absorbance spectra of seed and colloidal solution.

Powder of silver nanoparticles suspended in ethanol was undergone to laser light via optical fiber (approximately 2 min) to obtain a colloidal solution as one can see in Figure 1. With a laser energy of 0.5 μJ the solution changed its color from gray to yellow (Figure 3 inset). This means that the nanoparticles were disagglomerated, being that the yellow color is a characteristic of the silver colloidal solution with size particles between 6 and 28 nm [21,22]. The disagglomeration process was observed when the laser energy via optical fiber was up of 0.1 μJ. We assume that, the responsible mechanism of disagglomeration is the laser ablation. However, other kind of analysis must be done to fully understand this phenomenon.

Absorption curves of seed and colloidal solution are shown in Figure 3. Regarding the colloidal solution this exhibits a well-defined absorption peak at 406.5 nm whereas the seed solution exhibits a peak absorption at 408.3 nm and the absorption begins to increase for longer wavelength. The yellow hue is related to the laser power and the number of irradiating laser pulses on the solution, it also depends on the concentration of the solution.

The optical fiber was prepared by removing its coating, cleaving it and placing it into a colloidal solution to photodeposit silver nanoparticles on the optical fiber end as we can see in Figure 3. The laser energy provided by the optical fiber was 50 nJ with Gaussian profile. The laser power was continuously monitored and the laser was turned off when the nanoparticles provoked a loss of 1 dB (in a time less than 20 min). It is important to notice that if the laser is not turned on, there are not nanoparticles adhered on the optical fiber end. The silver nanoparticles begin to adhere to the optical fiber end, when the laser diode is turned on and the amount of nanoparticles adhered on the optical fiber end is related to the immersion time of the optical fiber into the colloidal solution.

**Figure 4.**
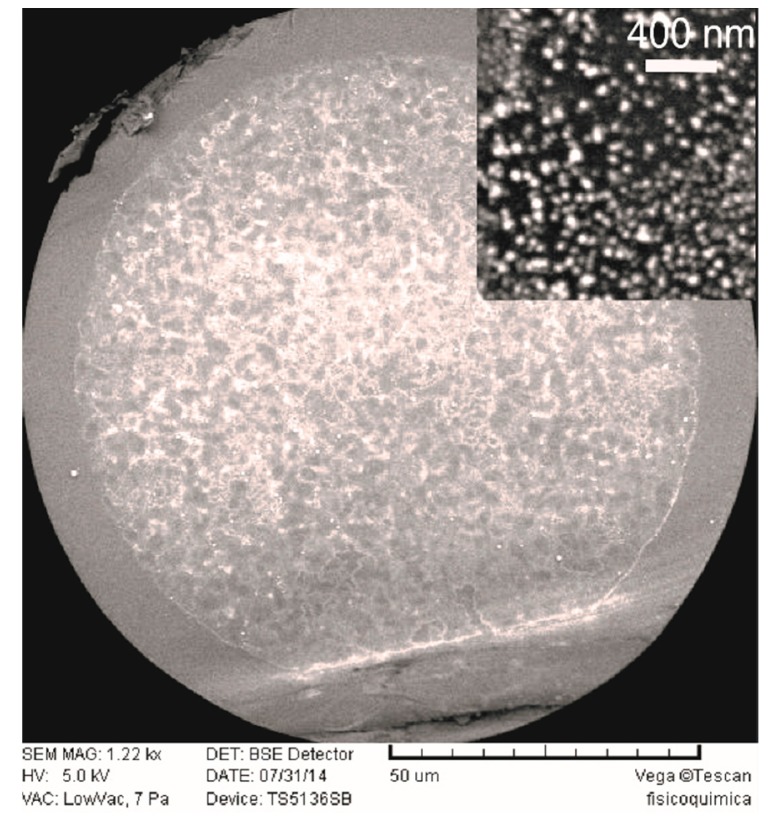
Image of the optical fiber end obtained with SEM.

An image of silver nanoparticles on the core of the optical fiber end obtained via a scanning electron microscope (TESCAN SEM Vega TS-5136SB) is shown in Figure 4. The inset is a magnification that shows silver nanoparticles agglomerated and disagglomerated whose sizes are less than 100 nm.

**Figure 5.**
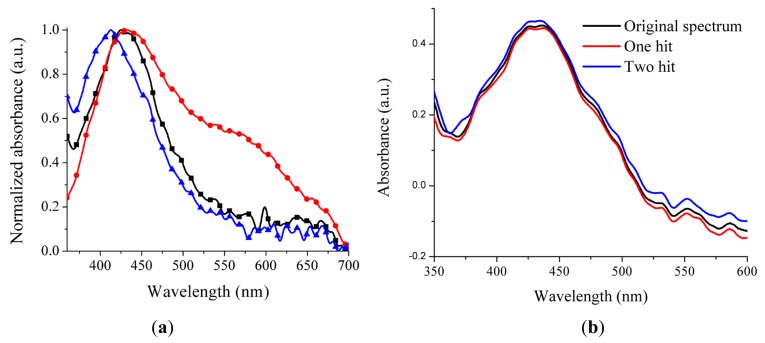
Sensor response based on LSPR localized in air. (**a**) The nanoparticles were photodeposited on the optical fiber end with the same conditions; (**b**) Sensor response before and after hitting the optical fiber.

The common way of characterizing the sensor response based on LSPR is through of the measurement of its extinction spectrum, which is defined as the sum of scattering and absorption spectra. The scattering cross-section, the absorption cross-section, and extinction cross-section depend on the silver nanoparticles dimensions. In the spectra transmission measurement (see Figure 2), both scattering and absorption of silver nanoparticles contribute to the total sensor response. The scattering cross-section dominates when the particle size is greater than 76 nm, whereas the absorption cross-section becomes dominated when the particle size is less than 44 nm [23]. Considering that, there are nanoparticles agglomerated and disagglomerated on the optical fiber end, both absorption and scattering spectra determine the sensor response. This response is shown in Figure 5, when the optical fiber sensor is localized in air. The LSPR peaks and their widths are different from each other as we can see in Figure 5a. These variations are provoked by the agglomeration of nanoparticles that affect the sensibilities and the experimental reproducibility. However, it is important to mention, that using the photodeposition technique with the same conditions is possible to obtain sensors with sensibilities between 40 and 67 nm/RIU.

The stability of nanoparticles adhered on the optical fiber end was verified by subjecting the optical fiber end to strong oscillations. We take the optical fiber below its tip (approximately 5 cm), then it was hardly hit with the index finger. The response of the optical fiber sensor before and after hitting the fiber is shown in Figure 5b. In this figure one can see that the LSPR spectrum did not change after having beaten the fiber. This means that nanoparticles adhered on the optical fiber end are stable.

**Figure 6.**
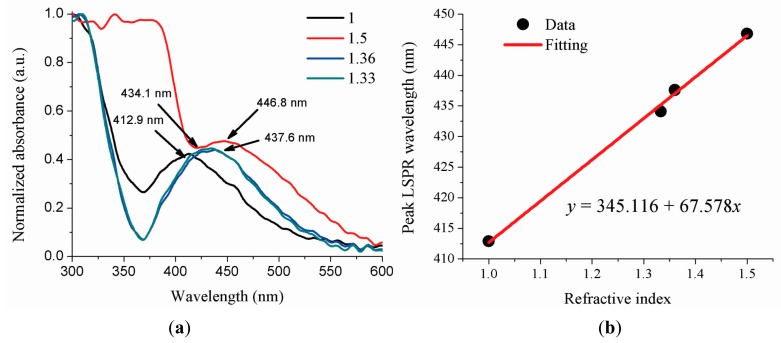
(**a**) Sensor response localized in air, deionized water, ethanol, and clove oil; (**b**) LSPR peak wavelength as a function of the refractive index.

The results using the optical fiber sensor based on LSPR when it is in air (*n* = 1.00), deionized water (*n* = 1.33), ethanol (*n* = 1.36), and clove oil (*n* ∼ 1.5) are shown in Figure 6. Normalized absorption spectrum as a function of refractive index changes are shown in Figure 6a, it can be seen that, there is a change (21.2 nm) in the LSPR peak wavelength position when the sensor was changed from air to deionized water. This position of the peak is moved to red when the refractive index of the aqueous medium is increased. Clove oil has a strong absorption in the ultraviolet region provoking that LSPR spectrum shape in this region has a high value. Figure 6b summaries the peak LSPR wavelength changes of the sensor with the change of refractive index, in which a linear curve can be fitted to the data. Therefore, it is possible to determinate the refractive index of an unknown aqueous media knowing the peak LSPR wavelength and using the Equation of the straight line that arises from the data (Figure 6b). Finally, from Equation 6 we can calculate the sensor sensitivity *S* = 67.6 nm/RIU.

## 4. Conclusions

We have implemented and characterized an optical fiber sensor based on LSPR phenomenon using silver nanoparticles. We have used a novel technique known as photodeposition to immobilize the silver nanoparticles on the optical fiber end in less than 20 min. This technique is low cost and simple instrumentation, furthermore it involves laser light which travels into the optical fiber and silver nanoparticles suspended in ethanol. Photodeposition technique has potential applications in this topic because of it is possible to photodeposit other kind of metallic nanostructures, for instance nanorods, nanowires, among others, on the optical fiber end and also to increase the sensitivity of the sensor. The optical fiber sensor instrumentation is very simple, it consists of a tungsten lamp, a spectrometer, and an optical fiber with nanoparticles. As expected, the response of this sensor is such that the LSPR peak wavelength shifts linearly to longer wavelengths as the refractive index increases, showing a sensitivity of 67.6 nm/RIU. The sensibility of our sensor is similar to others optical fiber sensors fabricated with self-assembled method. We think that is possible to increase the sensibility by optimizing the photodeposition process to prevent the formation of nanoparticles agglomerated. This optical sensor can be used for determining the quality in alcoholic beverages considering their refractive index.

## Figures and Tables

**Table 1.** Deposition methods used to fabricate optical fiber sensors based on localized surface plasmon resonance (LSPR).

**Deposition Method**	**Particle**	**Size Particle (nm)**	**LSPR Peak**	**Sensitivity**
Electron-beam lithography	Au nanodot	190	∼625 nm (in air)	∼195 nm/RIU [9,10]
	Au nanodisks	∼150	∼667 nm (in air)	226 nm/RIU [11]

Polyelectrolyte Self-assembly	Au	28	∼527 nm (in water)	71 nm/RIU [12]
		4	659 nm (in solution (*n* = 1.42))	13.09 AU/RIU [13]
		23	546 nm (in solution (*n* = 1.42))	5.85 AU/RIU [13]

Self-assembly	Au	47	∼550 nm (in solution (*n* = 1.33))	10.49 AU/RIU [14]
		8.4	1524.5 nm (in air)	−23.45 nm/RIU [15]
		24	∼570 nm (in solution (*n* = 1.33))	51 nm/RIU [16]
